# Characterization of Tissue Plasminogen Activator Expression and Trafficking in the Adult Murine Brain

**DOI:** 10.1523/ENEURO.0119-18.2018

**Published:** 2018-08-06

**Authors:** Tamara K. Stevenson, Daniel A. Lawrence

**Affiliations:** 1 Department of Molecular and Integrative Physiology, University of Michigan Medical School, Ann Arbor, MI 48109; 2 Department of Internal Medicine, Division of Cardiovascular Medicine, University of Michigan Medical School, Ann Arbor, MI 48109

**Keywords:** gene expression, interneuron, mossy fibers, protein-traffiking, somatostatin, tPA

## Abstract

Tissue plasminogen activator (tPA) is an immediate-early gene important for regulating physiological processes like synaptic plasticity and neurovascular coupling. It has also been implicated in several pathological processes including blood-brain barrier (BBB) permeability, seizure progression, and stroke. These varied reports suggest that tPA is a pleiotropic mediator whose actions are highly compartmentalized in space and time. The specific localization of tPA, therefore, can provide useful information about its function. Accordingly, the goal of this study was to provide a detailed characterization of tPA’s regional, cellular, and subcellular localization in the brain. To achieve this, two new transgenic mouse lines were utilized: (1) a PlatβGAL reporter mouse, which houses the β-galactosidase gene in the tPA locus and (2) a tPA^BAC^-Cerulean mouse, which has a cerulean-fluorescent protein fused in-frame to the tPA C-terminus. Using these two transgenic reporters, we show that while tPA is expressed throughout most regions of the adult murine brain, it appears to be preferentially targeted to fiber tracts in the limbic system. In the hippocampus, confocal microscopy revealed tPA-Cerulean (tPA-Cer) puncta localized to giant mossy fiber boutons (MFBs) and astrocytes in stratum lucidum. With amplification of the tPA-Cer signal, somatically localized tPA was also observed in the stratum oriens (SO)/alveus layer of both CA1 and CA3 subfields. Coimmunostaining of tPA-Cer and interneuronal markers indicates that these tPA-positive cell bodies belong to a subclass of somatostatin (SST)/oriens-lacunosum moleculare (O-LM) interneurons. Together, these data imply that tPA’s localization is differentially regulated, suggesting that its neuromodulatory effects may be compartmentalized and specialized to cell type.

## Significance Statement

The serine protease tissue plasminogen activator (tPA) has been shown to modulate numerous neurologic processes including synaptic plasticity and neurodegeneration. Many of the functional conclusions drawn about tPA activity, however, have not been affirmed by high-resolution, imaging analysis of tPA localization. To address these shortcomings, we used two new transgenic reporter mice to provide a detailed characterization of tPA expression in the adult murine brain. A comparison of these reporter mice demonstrates a differential expression pattern between the sites of tPA synthesis and its targeted localization in the hippocampus, amygdala, and basal ganglia. Moreover, colocalization and coexpression analysis reveals that tPA is primarily trafficked to presynaptic structures and that it’s predominant somatic and/or axonal localization is cell-type specific.

## Introduction

Tissue plasminogen activator (tPA) is a serine protease expressed in vascular endothelial cells with a well-established role in fibrinolysis. Biomechanistic understanding of tPA’s fibrinolytic function led to the development of recombinant tPA (rtPA) as a thrombolytic agent, and the current standard of care for moderate to severe ischemic stroke is thrombolytic therapy with rtPA (Prabhakaran et al., 2015). However, beyond 3–4.5 h following stroke onset, thrombolytic efficacy is diminished, and there is an increased risk of hemorrhagic conversion, limiting the therapeutic window for rtPA administration (Ahmed et al., 2010; Sandercock et al., 2012). The molecular mechanisms responsible for the increased risk of hemorrhage are thought, in part, to occur from exogenously administered rtPA crossing the ischemic, compromised blood-brain barrier (BBB) and acting through endogenous tPA-mediated signaling pathways on the abluminal side of the vasculature in the CNS to induce BBB opening (Su et al., 2008).

In addition to BBB regulation (Fredriksson et al., 2015) parenchymal brain tPA has been reported to be involved in other processes in the CNS, including neurite outgrowth (Krystosek and Seeds, 1981), regeneration (Akassoglou et al., 2000; Zou et al., 2006), synaptic transmission and synaptic plasticity (Wu et al., 2015; Frey et al., 1996; Huang et al., 1996), excitotoxic injury (Tsirka et al., 1995; Nicole et al., 2001; Siao et al., 2003), and neurovascular coupling (Park et al., 2008). One of the earliest studies implicating tPA in a non-fibrinolytic function found the serine protease to be an immediate-early gene that is upregulated in the hippocampus following seizures, kindling, and long-term potentiation (LTP; Qian et al., 1993), suggesting a proteolytic mechanism for activity-dependent structural changes at the synapse. Subsequent *in situ* hybridization studies showed tPA mRNA expression predominantly in hippocampal pyramidal and granule cell layers, and the granule cell layer of the cerebellum (Sappino et al., 1993).

While gross anatomic localization studies of tPA protein and protease activity have consistently shown tPA in the hilus and stratum lucidum layer of the hippocampus (Sappino et al., 1993; Salles and Strickland, 2002), more detailed cellular localization studies of tPA protein expression have been inconsistent (Fredriksson et al., 2015; Louessard et al., 2016). tPA-immunoreactivity, following colchicine treatment to block axo-dendritic transport, has been reported in glutamatergic cortical neurons and in the pyramidal and granule cell layers of the hippocampus (Louessard et al., 2016). In contrast, Fredriksson et al. (2015) primarily detected tPA-immunoreactivity in endothelial cells and a subset of perivascular interneurons. At the subcellular level, tPA appears to have a polarized distribution, as it has been localized to dense core vesicles in both presynaptic (Silverman et al., 2005; Scalettar et al., 2012) and postsynaptic (Lochner et al., 1998; Shin et al., 2004; Lochner et al., 2006) compartments; although these studies were done *in vitro* using clonal neuroendocrine cell lines or primary hippocampal neurons.

These disparate findings on localization of tPA have complicated and contributed to the multivariate hypotheses that exist regarding tPA’s function in the CNS. To address some of these discrepancies, we have used two transgenic mouse strategies: (1) a PlatβGAL reporter mouse, which has the β-galactosidase gene knocked-in to the tPA gene, *Plat*, and (2) a tPA^BAC^-Cerulean (tPA^BAC^-Cer) fusion mouse, which has a cerulean-fluorescent protein fused to tPA. The tPA^BAC^-Cer mice were generated using bacterial artificial chromosome (BAC) technology. Critically, large transgene vectors, like BACs, are more likely than smaller plasmids to produce copy-number dependent transgene expression, and thereby, recapitulate endogenous gene expression patterns (Van Keuren et al., 2009). In parallel analysis of coronal sections from PlatβGAL and tPA^BAC^-Cer mice, our results demonstrate that tPA’s protein localization is uncoupled from its site of synthesis. This differential expression pattern is most prominent in the hippocampus, but it is also pronounced in the amygdala and basal ganglia. Moreover, using high-resolution confocal microscopy, in the hippocampus we found tPA to be localized to giant mossy fiber boutons (MFBs) and astrocytes in stratum lucidum and somatically localized to interneurons in stratum oriens (SO)/alveus. Coexpression analysis indicates that these tPA-positive cell bodies in the hippocampus belong to a subset of somatostatin (SST)/oriens-lacunosum moleculare (O-LM) inhibitory interneurons. These results suggest that tPA is differently trafficked and positioned to have diverse modulatory effects on synaptic efficacy based on cell type and subcellular localization.

## Materials and Methods

### Transgenic mice

#### tPA^BAC^-Cerulean transgenic mice

Founder lines (863 and 876) for tPA^BAC^-Cer transgenic mice were generated using BAC technology. To generate tPA^BAC^-Cer transgenic mice, exon 14 of the tPA gene, *Plat*, on a 162.524 kb BAC acquired from chori.org (RP23-259A10), was replaced with a cerulean fluorescent gene (Rizzo et al., 2004) fused to the C-terminus of exon 14 of the murine tPA gene (NM_008872.2) followed by a bovine growth hormone polyadenylation signal sequence. The tPA-Cerulean (tPA-Cer) fusion gene is under control of the endogenous regulatory elements contained in the *Plat* locus. BAC DNA integrity was verified by restriction enzyme analysis via pulse field gel electrophoresis and exon sequencing before pronuclear microinjection of supraovulated eggs from (C57BL/6 x SJL)F1/TAC female mice. Transgenic mice were genotyped by PCR using primers that were specific to a remnant of the sub-cloning PGKneo vector and the tPA-Cer fusion gene (FWD 5’–CAT GAA GCA AGG ATC CAT GG–3’, and REV 5’–GGA ACT TCG CGG CCG CAG C–3’); and tPA protein expression was confirmed by analysis of brain homogenates from the founder lines. After PCR analysis of the cerulean fusion gene confirmed stable, germline transmission in F1 pups two founder lines, lines 863 and 876, were propagated; these mice were then backcrossed at least eight generations onto a C57BL/6J genetic background. Transgenic mice displayed normal gross anatomy and a Mendelian inheritance pattern.

#### PlatβGAL reporter mice

The PlatβGAL mice were acquired from the UC Davis Knockout Mouse Project (KOMP) Repository (Project ID: VG15085) on a C57BL/6NTac background. PlatβGAL mice were then backcrossed onto a C57BL/6J background for at least 10 generations. Per the KOMP Repository, PlatβGAL mice were generated by inserting a *LacZ*-containing targeting vector between exon 2 and 14 to produce a null allele. The insertion sites of the PlatβGAL mice were sequenced to confirm the appropriate insertion of the *LacZ* gene in the *Plat* locus.

All animals were housed in a controlled environment and were provided with food and water ad libitum. All animal experiments were approved by a local committee at the University of Michigan, and the studies were conducted in accordance with the United States Public Health Services Policy on Humane Care and Use of Laboratory Animals.

### Protein expression analysis

#### Sample preparation

Total tPA protein and enzymatic activity were analyzed using whole-brain homogenates from tPA^BAC^-Cer mice. The total and active tPA values from tPA^BAC^-Cer transgene-positive mice were normalized to transgene negative littermate controls for each experimental run. Two independent experiments were conducted for a combined total of six to seven mice per transgenic line. Briefly, brains were harvested into ice-cold extraction buffer [0.4 M HEPES, 0.1 M NaCl (pH 7.4), and 1% Triton X-100], homogenized for 1 min. (2 × 30 s) and centrifuged at 10,000 × *g* for 10 min. The supernatant was removed to a new, chilled 1.5-ml microcentrifuge tube and centrifuged again at 10,000 × *g* for 10 min. The supernatant was again removed to a new, chilled 1.5-ml microcentrifuge tube and used for ELISA, Luminex, and SDS-PAGE zymography assays.

#### ELISA

An ELISA was performed to measure tPA activity from brain tissue extracts. Briefly, avidin-coated microtiter plates (Molecular Innovations, AVI-PLATE) were incubated with a biotin-conjugated PAI-1 capture (1 μg/ml; Molecular Innovations, NTBIOCPAI) for 30 min at room temperature. After which, 100 μl of brain extract samples were loaded onto the plate and incubated for 1 h at room temperature. A rabbit anti-human tPA (3 μg/ml; Molecular Innovations, ASHTPA-GF) was used as the primary antibody and a donkey anti-rabbit HRP (1:5000; Jackson ImmunoResearch, 711-036-152) was used as the secondary. All sample and antibody incubations were followed by three washes of PBS-0.05% Tween 20. After the final wash, 3,3’,5,5’-tetramethylbenzidine (TMB) substrate (Molecular Innovations, TMB) was added to each sample for 3 min at room temperature. H_2_SO_4_ (1 N) was then added and the plate read on a spectrophotometer at 450 nm.

#### Luminex

To measure total murine tPA protein from tPA^BAC^-Cer brain extracts, 50 μg of Rabbit anti-murine tPA (mtPA; Molecular Innovations, ASMTPA-GF-HT) was coupled to Luminex carboxylated beads for mtPA capture. Standards of known concentration of murine tPA (Molecular Innovations, MTPA) and brain extract samples [diluted in 0.4 M HEPES, 0.1 M NaCl (pH7.4), and 1.0% Triton X-100] were loaded onto a 96 well filter plate (Millipore) and incubated with 5000 beads [PBS-1.0% bovine serum albumin (BSA)] for 2 h at room temperature in the dark. The solution from was removed from each well and washed twice with PBS-0.05% Tween 20. The beads were then mixed with continuous shaking in the dark at room temperature for 1 h with 2 μg/ml biotin-labeled rabbit anti-mouse tPA-high titer (Molecular Innovations, ASHTPA-HT), after which 10 μg/ml of Streptavidin, R-Phycoerythrin (ThermoFisher Scientific, S866) was added to each well for 1 h. The solution was removed from each well, and the beads were washed three times with PBS-0.05% Tween 20, and, lastly, sheath fluid was added for 5–10 min. The beads were then read with the Luminex 100 (medium setting; 10-μl sample size; 100 events/bead).

#### SDS-PAGE zymographies

Gel electrophoresis and zymography were performed as previously described (Huarte et al., 1985). Briefly, 1 μg of protein from homogenized whole-brain tissue extracts from transgene-positive and transgene-negative tPA^BAC^-Cer mice were loaded onto an in-house prepared 10% polyacrylamide gel with plasminogen ([10.0 μg/ml]_FINAL_) and casein ([1.0 mg/ml]_FINAL_). Samples were run for 30 min at 100 V through the stacking gel and 200 V for 40 min through the running gel. Gel was washed 4 × 30 min in 2.5% Tx-100 (dH_2_O) and then briefly washed for 5 min in 0.1 M Tris buffer (pH 8.1) before developing in 0.1 M Tris buffer at 37°C for 4 h. Gels were stained with Bio-Safe Coomasie (Bio-Rad, 1610786); bands devoid of stain indicate areas of proteolytic activity.

### Immunofluorescence and histochemical analysis

#### Immunofluorescence analysis

Mice were anesthetized with isoflurane and sacrificed by transcardiac perfusion for 3 min with PBS followed by perfusion for 5 min with 4% paraformaldehyde (PFA). Brains were harvested and postfixed in 4% PFA for 1 h at 4°C, then overnight in PBS. The brains were then moved to a 30% sucrose solution and kept at 4°C till submerged. Subsequently, dorsal hippocampal sections (14 and 50 μm) and serial sections (14 μm, bregma +1.0 to bregma -8.0) were cut coronally for immunofluorescence analysis of tPA expression. When using the Rabbit anti-mtPA antibody, sections underwent antigen retrieval (DAKO, S1700); the additional antigen retrieval step was not necessary for other antibodies. Sections were permeabilized with 0.50% Triton X-100 (PBS) for 20 min at room temperature and blocked in 3% BSA (PBS) for 1 h at room temperature. The sections were then incubated with primary antibodies in 2% BSA (PBS) overnight at 4°C, followed by incubation with secondary antibodies in 2% BSA (PBS) for 1 h at room temperature. When using biotin-conjugated primary antibodies and their respective streptavidin-conjugated secondary was used, a biotin-blocking kit was used to reduce background (ThermoFisher Scientific, E21390). For amplification using the Tyramide SuperBoost kit (ThermoFisher Scientific, B40932) detection protocols were followed according to the manufacturer’s instructions.

The primary antibodies used were as follows: calbindin D28K (rabbit anti-calbindin, 1:500; Synaptic Systems, 214002; lot #214002/3), microtubule-associated protein 2 (rabbit anti-MAP2, 1:1000; Millipore, AB5622; lot #2624211), zinc transporter 3 (guinea pig anti-ZnT3, 1:500; Synaptic Systems, 197004; lot #197004/4), excitatory amino acid transporter 2 (rabbit anti-EAAT2, 1:500; Synaptic Systems, 250203; lot #250203/3), GFP (chicken anti-GFP, 1:1000; abcam, ab13970; lot #GR236651-7 and GR236651-14), NeuN (guinea pig anti-NeuN, 1:400; Synaptic Systems 266004; lot #266004/2-14 and 266004/7), GAD65 (guinea pig anti-GAD65, 1:500; Synaptic Systems 198104; lot #198104/7), murine tPA (rabbit anti-mtPA, 12 μg/ml; Molecular Innovations, ASMTPA-GF-HT; lot #804 and 914), metabotropic glutamate receptor type 1a (rabbit anti-mGluR1a, 1:200; Sigma, G9665; lot #SLBL4165V), SST (rat anti-SST, 1:100; Millipore, MAB354; lot #2885355, 3005269), CD31 (rat anti-mCD31, 1:100; BD Biosciences, 550274; lot #21055). The secondary antibodies used were as follows: biotin-conjugated goat anti-chicken IgY H&L (1:100; abcam, ab6876), goat anti-guinea pig IgG (H + L) 568 (1:500; ThermoFisher Scientific, A-11075), donkey anti-rabbit IgG (H + L) 568 (1:500; ThermoFisher Scientific, A-10042), donkey anti-guinea Pig IgG (H + L) 594 (1:500; Jackson ImmunoResearch, 706-585-148), donkey anti-rat IgG (H + L) 594 (1:500; ThermoFisher Scientific, A-21209), donkey anti-rabbit IgG (H + L) 594 (1:500; ThermoFisher Scientific, A-21207), tyramide-conjugated Alexa Fluor 488 (ThermoFisher Scientific, B40953). The sections were mounted using VectaShield anti-fade mounting medium (Vector Laboratories, H-1000).

#### 5-Bromo-4-chloro-3-indolyl-β-D-galactoside analysis

Heterozygous, homozygous, and wild-type PlatβGAL mice (mice, *n* = 3–5 per genotype) were used to examine the regional somatic expression of tPA. Homozygous PlatβGAL mice, which are null for tPA with two copies of the β-Gal gene, served as control mice for immunohistochemical stains that used an antibody directed against mtPA. PlatβGAL mice were anesthetized with isoflurane and sacrificed by transcardiac perfusion for 3 min with PBS and 1 min with 2% PFA, and postfixed in 2% PFA for 1 h at 4°C. Brains were then cryopreserved and sectioned by microtome. Dorsal hippocampal sections and serial sections (50 μm, bregma +1.0 to bregma -8.0) were stained and analyzed for LacZ reporter gene expression using the β-Galactosidase Reporter Gene Staining kit (Sigma, GALS).

### Image acquisition, processing, and analysis

#### Widefield and confocal microscopy

For PlatβGAL and tPA^BAC^-Cer transgenic mice, low-resolution images were acquired on an inverted Nikon Te2000 widefield microscope equipped with a MicroPublisher 5.0 RTV color camera and a CoolSNAP HQ2 CCD camera or an inverted Ti Nikon widefield microscope with an ANDOR Zyla sCMOS camera. High-resolution fluorescent images of the dorsal hippocampus in tPA^BAC^-Cer transgenic mice were taken using an upright confocal laser scanning microscope (Leica SP5X). The SP5X is equipped with an acousto-optical beam splitter (AOBS) and a tunable white-light laser; accordingly, the following Ex/Em combinations were used: Cerulean (458/468-558) and Alexa Fluor 568 (568/578-720); and Alexa Fluor 488 (488/498-584) and Alexa Fluor 594 (594/604-750). Images were acquired with a 20× multi-immersion objective or a 63× oil objective [Plan-Apo, 1.4 numerical aperture (NA)] at a scanning rate of 200 Hz with 4× line averaging at 2× or 4× optical zoom. Each frame consists of 512 × 512 pixels or 1024 × 1024 pixels. Z-stacks were collected at 0.5- or 1-μm increments, ranging in total thickness from 5.0 to 35 μm, respectively, with the pinhole set to 1 Airy unit.

#### Image processing and colocalization analysis

Widefield images (4×, 10×, 20×, or 40× objectives) of hippocampal and serial sections from PlatβGAL mice and tPA^BAC^-Cer transgene-positive and transgene-negative mice were stitched using MetaMorph Image Analysis software or Nikon’s NIS-Elements Advanced Research software package, respectively. Further processing was done using the open source image processing package FIJI (Schindelin et al., 2012). Confocal images are presented as either maximum intensity projections, orthongonal slices, or 3D maximum projections using FIJI’s 3D viewer (Schmid et al., 2010).

Subcellular colocalization analysis of tPA-Cer puncta in stratum lucidum was performed using the JACoP (Just Another Colocalization Plugin) analysis software in FIJI (Bolte and Cordelières, 2006). Images were concatenated from 5-μm z-stacks (Δz = 0.5 μm) that were independently acquired two to four times (ZnT3: mice *n* = 10, lines 863 and 876; EAAT2: mice *n* = 6–8, lines 863 and 876, respectively; MAP2: mice *n* = 4, lines 863 and 876). Manders coefficient and Costes randomization control were used to quantify tPA-Cer colocalization (Costes et al., 2004; Bolte and Cordelières, 2006; Dunn et al., 2011). The Manders coefficient was chosen because it is a more sensitive measure of colocalization for partial colocalization events and when there are large differences in fluorescent intensity between fluorophores (Bolte and Cordelières, 2006; Dunn et al., 2011). The Manders coefficient, which does not mathematically take into account average fluorophore intensity values, ranges from 0 to 1, with 0 corresponding to no overlap and 1 to complete overlap. Two coefficients are given: M1 and M2, where M1 is the summed intensities of fluorophore 1 that are coincident with fluorophore 2, divided by the total intensity of fluorophore 1; M2 is calculated the same but for fluorophore 2. Costes randomization control provides a statistical assessment of whether or not observed colocalization events could be expected to occur by chance. It is calculated by comparing the coincidence of colocalization in an original image against the coincidence of colocalization in a randomized imaged of shuffled pixels (200 times). Costes approach is expressed as a percentage; a probability *p* value of ≥95% suggests that colocalization is significant and not random (Costes et al., 2004).

Cell count of tPA-Cer and SST-positive cell bodies was performed in the dorsal hippocampus of tPA^BAC^-Cer transgenic mice over an approximate 500-μm range from -2.0 to -2.5 bregma. tPA-Cer and SST-positive cells were counted manually using the ROI Manager in FIJI. Cells were deemed positive if their mean pixel intensity was 1 SD above the mean pixel intensity for the image (Liao et al., 2016). Cell count data were gathered from two to four hippocampal sections per mouse (mice, *n* = 8) for each of tPA^BAC^-Cer transgenic lines and their transgene negative littermate controls. Cell count data were averaged per mouse and statistics were generated using GraphPad Prism, version 7.0. Data are presented as the mean ± 95% confidence interval [CI_(0.95)_] of tPA-Cer-expressing cells from SO/alveus (CA1 and CA3, respectively) and stratum radiatum (SR)/stratum pyramidale (SP; CA1 and CA3). The percentage mean ± CI_(0.95)_ of tPA-Cer cells that coexpress SST and the percentage mean ± CI_(0.95)_ of SST-positive cells that coexpress tPA-Cer is also given. Immunohistochemical analysis of tPA-Cer cell bodies that coexpress markers of O-LM interneurons was gathered from stainings from four to eight mice per coexpression marker.

#### Experimental design and statistical analysis

Experimental design, including all critical variables for independent replication, is described in detail in the Materials and Methods for each experiment. Briefly, for all analysis using PlatβGAL and tPA^BAC^-Cer transgenic mice, a mixture of adult male and female mice was used (age, 12–45 weeks). Wild-type PlatβGAL littermates were used as controls for β-Gal stains, while homozygous PlatβGAL littermates were used as controls for immunoreactivity against tPA. When evaluating global protein expression or using antibodies directed against GFP, tPA^BAC^-Cer transgene negative littermates were used as controls. Brightness and contrast were adjusted over the entire image and applied equally to control images from transgene negative samples for figure display. All image processing and statistical analysis of colocalization was preformed using the JACoP software in FIJI and described in detail in Materials and Methods. Statistical *t* tests were performed in GraphPad Prism, version 7.0, and a significance criterion of *p* < 0.05 was adopted. All other graphs and statistics [including mean, SEM, and CI_(0.95)_] were also generated using GraphPad Prism.

## Results

### Global and regional expression pattern of tPA in the adult murine brain

**Figure 1.**
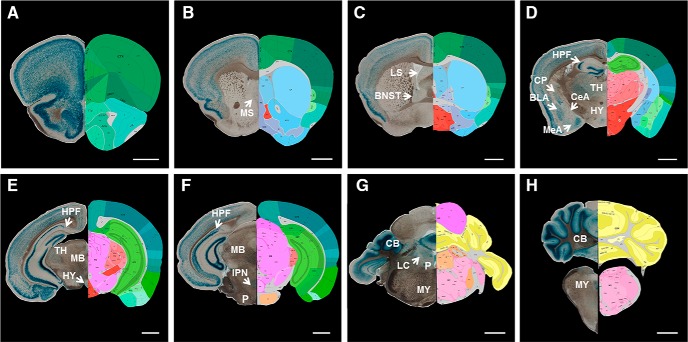
5-Bromo-4-chloro-3-indolyl-β-D-galactoside analysis of tPA expression in PlatβGAL reporter mice. Representative images (10×) from a heterozygous PlatβGAL reporter mouse (mice, *n* = 5) stained for β-Gal. Sections (50 μm) were cut coronally starting from the frontal cortex around bregma +2.5 and progressing caudally to the cerebellum around bregma -8.0. tPA/β-Gal activity is strongly present in the cortex (***A–F***), in the granule and pyramidal cell layers of the hippocampus (***D–F***), and in the molecular and granular layers of the cerebellum (***G***, ***H***). More diffuse tPA/β-Gal staining is observable in subcortical regions, such as the medial (***B***) and lateral septal nuclei (***C***), the bed nucleus of the stria terminalis (***C***), caudate/putamen (***D***), the basolateral and centromedial nuclei of the amygdala (***D***), and thalamus and hypothalamus (***D***, ***E***). The locus coeruleus, where neuroserpin, the neuronal inhibitor of tPA is highly concentrated, is largely devoid of tPA/β-Gal staining (***G***). There are also distinct β-Gal clusters in midbrain, pontine, and medulla structures (***F***, ***G***), such as the interpeduncular nucleus (***F***). Coronal reference atlas images (were taken from the Allen Developing Mouse Brain Atlas; http://mouse.brain-map.org/static/atlas). From ***A****–****H***, the following thumbnails were used: 32, 47, 53, 71, 82, 89, 109, and 123, respectively. MS, medial septal nuclei; LS, lateral septal nuclei; BNST, bed nucleus of the stria terminalis; HPF, hippocampal formation; TH, thalamus; CP, caudate/putamen; BLA, basolateral nucleus of the amygdala; CeA, central nucleus of the amygdala; MeA, medial nucleus of the amygdala; HY, hypothalamus; MB, midbrain; IPN, interpeduncular nucleus; P, pons; LC, locus coeruleus; CB, cerebellum; MY, medulla. Scale bars: 1 mm. Reference atlas images credit: Allen Institute.

**Figure 2.**
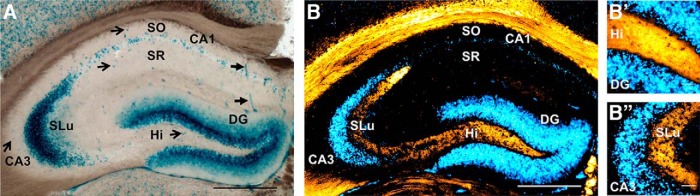
Immunohistochemical analysis of tPA protein expression in the hippocampus of PlatβGAL reporter mice reveals a differential expression pattern between the sites of tPA synthesis and tPA trafficking. ***A***, Representative image (10×) of a 50-μm hippocampal section from a heterozygous PlatβGAL reporter mouse (mice, *n* = 5). tPA is shown to be strongly expressed in the granule cell layer of the dentate gyrus and the pyramidal cell layer of hippocampal regions CA1–CA3. There are also scattered β-Gal puncta (arrows) in the hilus, SR, and SO; β-Gal staining is also present in blood vessels in the hippocampus (filled arrows). ***B***, Immunohistochemical analysis of a representative image (10×) from a 50-μm hippocampal section from a heterozygous PlatβGAL reporter mouse (mice, *n* = 5) using antibodies directed against murine tPA (orange). In contrast to the β-Gal stain (cyan), tPA is not expressed in the cell body layers, but in the mossy fiber axons of dentate granule cells in the hilus and stratum lucidum lamina (***B’***, ***B’’***, 40×). To visualize the colored β-Gal stain in the immunofluorescent image captured showing tPA-immunoreactivity, a negative of the fluorescent image was generated and pseudo-colored cyan. SLu, stratum lucidum; Hi, hilus; DG, dentate gyrus. Scale bars: 500 μm (***A***, ***B***) and 50 μm (***B’***, ***B’’***).

PlatβGAL reporter mice were used to characterize the global expression pattern of tPA in the adult murine brain. Serial coronal sections (50 μm) from heterozygous PlatβGAL mice were stained for β-Gal to assess regional patterning of somatic tPA expression. Prominent staining is present in layers 2-6 of the cortex (Fig. 1*A–F*), with an especially strong β-Gal signal highlighting the compact granule cell layer of the dentate gyrus and the pyramidal cell layer of the dorsal hippocampal CA3 subfield (Fig. 1*D*). Although less concentrated, the pyramidal cell layer of the CA1 and CA2 subfields in the dorsal hippocampus also demonstrates tPA/β-Gal expression (Fig. 2*A*). In addition, β-Gal staining is apparent in blood vessels throughout the adult murine brain, as illustrated in the hippocampal formation (Fig. 2*A*, filled arrows). More diffuse reporter gene expression is present in subcortical regions, like the medial (Fig. 1*B*) and lateral (Fig. 1*C*) septal nuclei, the bed nucleus of the stria terminalis (Fig. 1*C*), the thalamus and hypothalamus (Fig. 1*D*,*E*), caudate/putamen (Fig. 1*D*), and the basolateral and centromedial nuclei of the amygdala (Fig. 1*D*), while intense β-Gal staining populates the molecular and granular layers of the cerebellum (Fig. 1*G*,*H*). There is also noticeable β-Gal staining in the midbrain, pons, and medulla (Fig. 1*E–H*); specifically, there is a cluster of reporter gene expression in the interpeduncular nucleus (Fig. 1*F*). The interpeduncular nucleus is an integral group of cells involved in limbic midbrain circuitry and has been implicated in active avoidance behavior (Hammer and Klingberg, 1990). Interestingly, multiple groups have demonstrated a role for tPA in avoidance behavioral tasks (Huang et al., 1996; Calabresi et al., 2000; Pawlak et al., 2002), although interpeduncular tPA has never been explicitly examined. Also, in the pontine central gray (Fig. 1*G*), there is a small area devoid of β-Gal staining that appears to correspond to the locus coeruleus, which is known for its high concentration of neuroserpin, the neuronal inhibitor of tPA (Krueger et al., 1997). The regional patterning of tPA in the PlatβGAL mice is largely consistent with previous reports examining tPA expression using [^P^32]-labeled tPA cRNA probes (Sappino et al., 1993) and transgenic mice with tPA promoter-directed expression of β-Gal (Carroll et al., 1994; Yu et al., 2001). However, some differences were observed, such as strong tPA expression in the CA3 subfield and blood vessels (Fig. 2*A*), suggesting that the earlier studies lacked either the resolution or specific regulatory elements important for regional and cell specific expression.

### Differential expression pattern between where tPA is synthesized and where it is trafficked in the dorsal hippocampus

Immunohistochemical analysis of heterozygous PlatβGAL mice, which have one functional copy of the tPA gene and one copy of the LacZ gene, reveals tPA to have a differential expression pattern. Using an antibody directed against tPA, there is a distinct uncoupling between the sites of tPA synthesis and sites of tPA trafficking. This is most apparent in the dorsal hippocampus where the granule cell layer of the dentate gyrus is brightly positive for β-Gal, but devoid of tPA-immunoreactivity (Fig. 2*A*,*B*,*B’*). Conversely, the mossy fiber axonal tracts of the granule cells that project into the hilus (Fig. 2*B’*) and traverse along stratum lucidum (Fig. 2*B’’*) are strongly immunoreactive against anti-tPA antibodies. Although strongly expressing β-Gal, no tPA-immunoreactivity is detectable in the granule cell layer or CA3 pyramidal layer (Fig. 2*B–B’’*).

**Figure 3.**
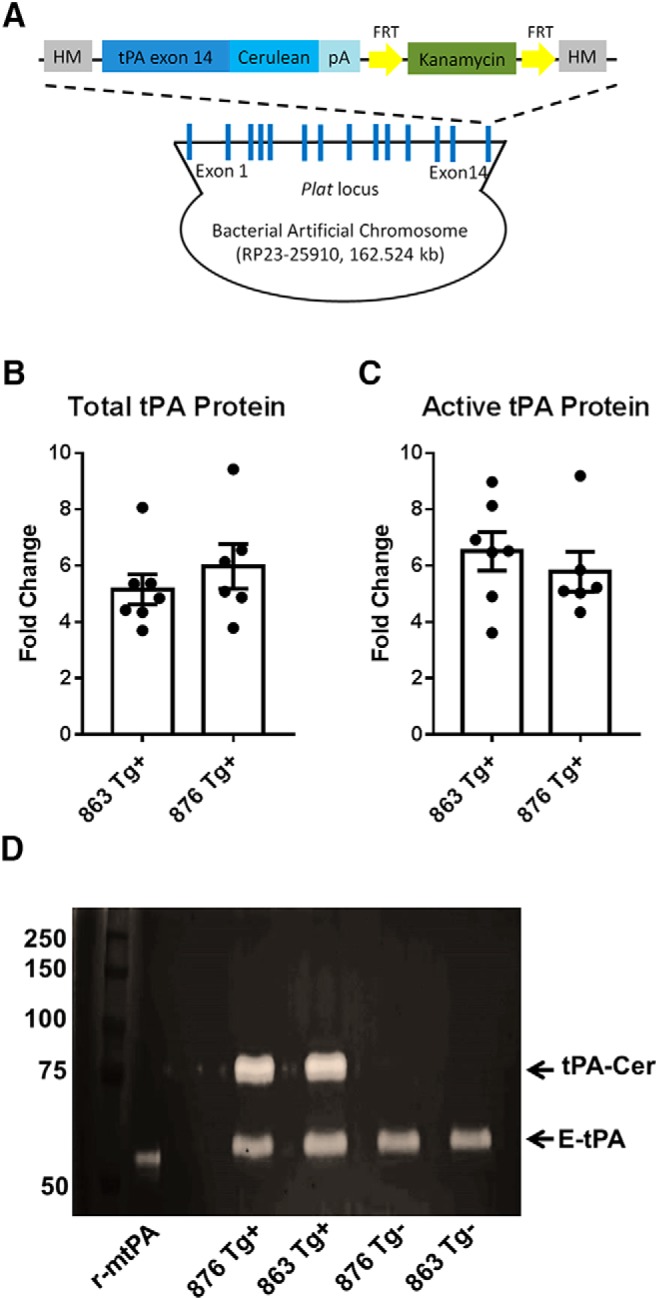
Generation and global tPA protein characterization of tPA^BAC^-Cerulean transgenic mice. ***A***, Founder lines for tPA^BAC^-Cer transgenic mice (863 and 876) were generated using BAC technology. To generate tPA^BAC^-Cer transgenic mice, a kanamycin-resistance recombineering cassette containing a cerulean fluorescent gene fused to the C-terminus of exon 14 of the tPA gene was recombineered into a BAC. The tPA-Cer fusion gene is under control of endogenous regulatory elements contained in the *Plat* locus. ***B****–****D***, Protein expression profile of tPA^BAC^-Cer transgenic mice. Brains from transgene-positive and transgene-negative adult tPA^BAC^-Cer mice were harvested and homogenized for total and active protein quantification using a bead-based Luminex assay (***B***) and ELISA (***C***). Data are presented as the mean ± SEM fold change from tPA protein levels in transgene negative littermate controls (mice *n* = 6-7). No statistical difference was noted in total and active tPA protein levels between line 863 and 876. ***D***, Zymographic analysis of whole-brain homogenates from tPA^BAC^-Cer transgene positive and negative mice (lines 863 and 876) visually delineates endogenous tPA (lower molecular weight bands, E-tPA) and the tPA-Cer from the BAC (higher molecular weight bands, tPA-Cer). Not only is there increased levels of tPA protein in both tPA^BAC^-Cer transgenic lines, but the tPA-Cer protein is proteolytically active. FRT, flippase recognition target; HM, homology arms; r-mtPA, recombinant-murine tPA; E-tPA, endogenous-tPA.

Given the disparate expression profiles of where tPA is synthesized and where it is trafficked, a more targeted strategy for visualizing tPA is needed. To achieve this, BAC technology was used to generate transgenic fusion reporter mice that have a fluorescent cerulean protein tagged to the C-terminus of tPA (Fig. 3*A*). Compared to a reporter gene approach, the tPA-Cer fusion reporter approach facilitates a more precise analysis of the regional, cellular, and subcellular expression pattern of tPA in the adult murine brain. And, it can lead to greater functional insights about the dynamic nature of tPA in the brain, including cellular packaging and transport, cellular communication, and regional connectivity.

### Global tPA protein expression profile in tPA^BAC^-Cerulean transgenic mice

Before a detailed characterization of tPA localization in tPA^BAC^-Cer mice, global tPA protein expression levels were measured. Brains from transgene-positive and transgene-negative adult tPA^BAC^-Cer mice (mice, *n* = 6–7) were harvested and homogenized for total and active protein levels using a bead-based Luminex assay (Fig. 3*B*) and ELISA (Fig. 3*C*). tPA^BAC^-Cer mice were found to have an approximate 5–6 fold change in total tPA levels from transgene negative mice (Fig. 3*B*), and an approximate 6 fold change in active tPA levels from transgene negative mice (Fig. 3*C*). No statistical difference in total or active tPA was noted between the two transgenic lines. To more specifically discriminate between endogenous tPA protein and that which is from the BAC, whole-brain homogenates from transgenic tPA^BAC^-Cer mice (lines 863 and 876) and their respective transgene negative littermate controls were run on a zymography gel (Fig. 3*D*). Transgene-positive tPA^BAC^-Cer mice showed enzymatic activity from both endogenous tPA (∼60 kDa) and the tPA-Cer protein (∼75 kDa). Samples from transgene negative mice did not display the higher molecular weight band that is indicative of tPA protein with the added cerulean fluorescent protein.

### tPA-Cer fusion protein is prominently expressed in limbic structures and blood vessels in the adult murine brain

**Figure 4.**
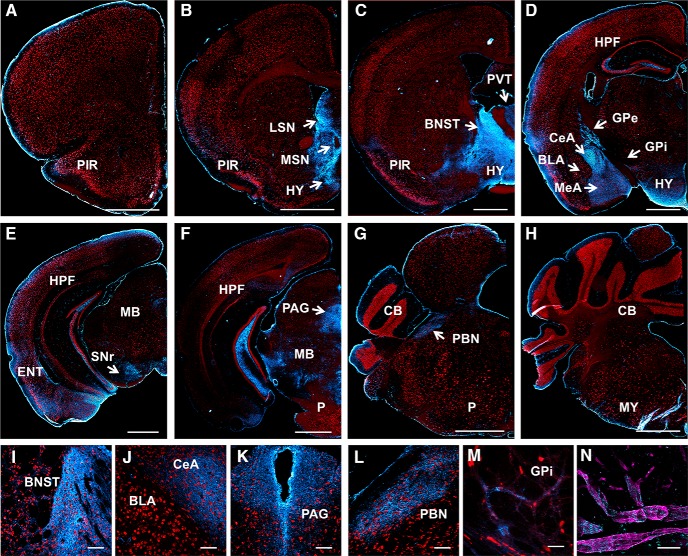
tPA-Cer fusion protein is prominently expressed in limbic structures and blood vessels in the adult murine brain. Images shown are representative of stitched serial coronal sections from tPA^BAC^-Cer transgenic mice (*n* = 3) captured on a widefield microscope (***A****–****H***, 10×; ***I****–****L***, 20×; ***M***, 40×) or confocal microscope (***N***, 63×). Cryosections stained for the neuronal marker NeuN (red) clearly distinguishes the faint tPA-Cer (cyan) cells bodies observed in the piriform (***A****–****D***) and entorhinal cortex (***E***), and the tPA-Cer fluorescent nerve fibers found in the medial and lateral septal nuclei (***B***), the bed nucleus of the stria terminals (***C***, ***I***), the paraventricular nucleus of the thalamus (***C***) and hypothalamus (***D***), the central (***D***, ***J***) and medial (***D***) nuclei of the amygdala, the external globus pallidus (***D***) and internal globus pallidus (***M***) of the basal ganglia, the substantia nigra pars reticulata, (***E***), the periaqueductal gray (***F***, ***K***), and the parabrachial nucleus (***G***, ***L***). tPA is also robustly expressed in the hilus and mossy fiber pathway of the hippocampus (***D****–****F***). ***G***, ***H***, In contrast to the PlatβGAL reporter mice, tPA expression is not observable in the cerebellum. ***N***, Brightly positive tPA-Cer puncta are noticeable throughout all brain regions in blood vessels using the endothelial cell marker, CD31 (magenta). PIR, piriform cortex; ENT, entorhinal cortex; LS, lateral septal nuclei; MS, medial septal nuclei; BNST, bed nucleus of the stria terminalis; PVT, paraventricular nucleus of the thalamus; HY, hypothalamus; HPF, hippocampal formation; CeA, central nucleus of the amygdala; MeA, medial nucleus of the amygdala; BLA, basolateral nucleus of the amygdala; PAG, periaqueductal gray; PBN, parabrachial nucleus; GPe, globus pallidus external segment; GPi, globus pallidus internal segment; SNr, substantia nigra pars reticulata; MB, midbrain; P, pons; CB, cerebellum; MY, medulla. Scale bars: 1 mm (***A–H***), 100 μm (***I–M***), and 50 μm (***K***, ***N***).

**Figure 5.**
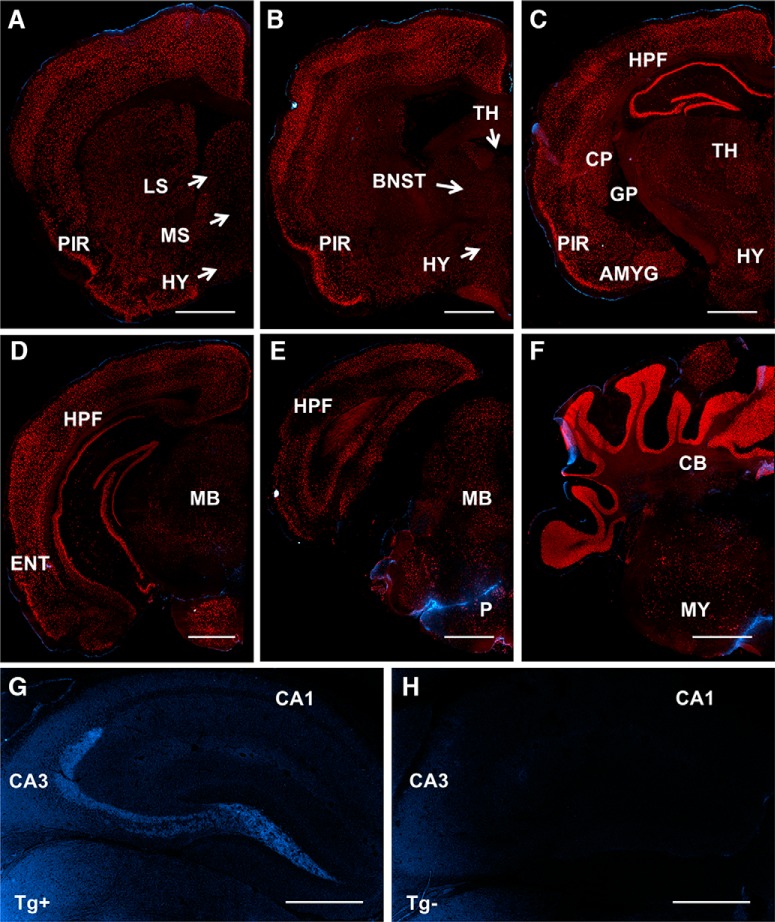
tPA-Cer fluorescence is not observed in tPA^BAC^-Cer transgene negative littermate controls. ***A****–****D***, Images shown are representative of stitched serial coronal sections from tPA^BAC^-Cer transgene negative littermate mice (*n* = 2) captured on a widefield microscope (10×). Cyrosections (14 μm) stained for the neuronal marker NeuN (red) show no cerulean fluorescence in cell bodies in the piriform (***A****–****C***) or entorhinal cortex (***D***). Cerulean fluorescence is also not observable in the medial and lateral septal nuclei (***B***), the bed nucleus of the stria terminals (***B***), the amygdala (***C***), globus pallidus (***C***), or the thalamus and hypothalamus (***B***, ***C***). Midbrain and pontine brain structures are devoid of any cerulean fluorescence (***D***, ***E***), as are the medulla and cerebellum (***F***). ***G***, ***H***, Representative images from widefield microscopy (4×) of the hippocampus from a tPA^BAC^-Cer transgene-positive (Tg+) mouse and its transgene-negative (Tg-) littermate. Cerulean fluorescence is clearly observable in the mossy fiber pathway of a tPA^BAC^-Cer transgene-positive mouse, but completely absent in the transgene-negative control. Cerulean fluorescent artifacts from edge effects or folds are apparent in panels ***C***, ***E***, ***F***. PIR, piriform cortex; ENT, entorhinal cortex; LS, lateral septal nuclei; MS, medial septal nuclei; BNST, bed nucleus of the stria terminalis; TH, thalamus; HPF, hippocampal formation; AMYG, amygdala; HTH, hypothalamus; CP, caudate/putament; GP, globus pallidus; MB, midbrain; P, pons; CB, cerebellum; MY, medulla. Scale bars: 1 mm (***A–F***) and 500 μm (***G***, ***H***).

In a global survey of tPA-Cer fluorescence in tPA^BAC^-Cer transgenic mice, tPA-Cer puncta appear to be primarily restricted to two pools: nerve fibers (Fig. 4*A–G*,*I–M*) and vascular endothelial cells (Fig. 4*N*). No observable cerulean fluorescence was detected in transgene negative littermate controls (Fig. 5*A–F*,*H*). In the brain parenchyma, faint tPA-Cer cell bodies are noticeable in the piriform and entorhinal cortex (Fig. 4*A–E*), while more predominant and intense tPA-Cer fluorescence is seen in nerve fibers in hippocampal and subcortical regions of the brain. Juxtaposed to the somatic neuronal marker NeuN, tPA-Cer fluorescence is clearly not localized to the cell body; rather, it appears to be expressed in nerve fibers emanating or innervating brain structures associated with the limbic system, including the medial and lateral septal nuclei (Fig. 4*B*), the bed nucleus of the stria terminalis (Fig. 4*C*,*I*), the paraventricular nucleus of the thalamus (Fig. 4*C*), hypothalamus (Fig. 4*C*,*D*), the mossy fiber pathway of the hippocampus (Figs. 4*D–F*, 5*G* ), the centromedial nucleus of the amygdala (Fig. 4*D*,*J*), the external globus pallidus (GPe) and internal globus pallidus (GPi) nuclei of the basal ganglia (Fig. 4*D*,*M*), the substantia nigra pars reticulata (SNr; Fig. 4*E*), the periaqueductal gray (Fig. 4*F*,*K*), and the parabrachical nucleus (Fig. 4*G*,*L*).

*In situ* zymography previously demonstrated tPA activity in the mossy fiber pathway and hypothalamus (Sappino et al., 1993), the bed nucleus stria terminalis (Matys et al., 2004), and the centromedial, but not basolateral, nucleus of the amygdala (Pawlak et al., 2003). To our knowledge, however, we are the first to report on tPA expression in the paraventricular nucleus of the thalamus, the periaqueductal gray, and the parabrachial nucleus. These regions, in addition to the bed nucleus stria terminalis and the hypothalamus, are connected via afferent and/or efferent projections to the centromedial amygdalar nucleus (Janak and Tye, 2015; Penzo et al., 2015; Tasan et al., 2016; Babaev et al., 2018). Neurons in the basolateral nucleus also send projections to the centromedial nucleus. And, while β-Gal expression was detected in both the basolateral and centromedial nuclei, given the complex circuitry of the amygdala, it is unclear if the tPA-Cer fluorescence in the centromedial nucleus is trafficked tPA from basolateral nerve projections or trafficked tPA in afferent/efferent nerve fibers to/from other brain regions.

We also report, for the first time, on tPA expression in the GPe and GPi nuclei of the basal ganglia. Indeed, the differential expression of somatic tPA/β-Gal and trafficked tPA-Cer is appreciable when comparing tPA expression in the PlatβGAL (Fig. 1*D*) and tPA^BAC^-Cer (Fig. 4*D*) transgenic mice. While β-Gal staining is present in the caudate/putamen nucleus, it is devoid in both the GPe and GPi nuclei in the PlatβGAL reporter mice (Fig. 1*D*). In contrast, tPA-Cer fluorescence is absent in the caudate/putamen, but present in the GPe, GPi, and SNr (Fig. 4*D*,*E*). The GPi and SNr are equivalent anatomic structures, both embryologically and functionally (Purves et al., 2001), as they are the output nuclei of the basal ganglia. Given that tPA-Cer, but not β-Gal, is present in the GPe, it is likely that the observed tPA-Cer is part of the direct loop through the basal ganglia. The circuitry of the direct loop involves GABAergic neurons that project from caudate/putamen through the GPe to the GPi or GABAergic neurons from caudate/putamen that travel through the strionigral fibers to SNr (Purves et al., 2001; Gilman and Newman, 2002). In turn, both the GPi and SNr send GABAergic projections to the thalamus. The direct loop is known to increase thalamocortical excitation, and it is important for the selection of desired behaviors (Purves et al., 2001; Gilman and Newman, 2002). Together, the high expression of tPA-Cer fluorescence in cell bodies and nerve fibers in limbic structures, especially amygdalar-associated brain regions, strongly support a role for tPA in affective, motivational, and anxiety-like behavior (Pawlak et al., 2002, 2003; Matys et al., 2004). And, while tPA has yet to be studied in the basal ganglia, more recent evidence has suggested that, functionally, the basal ganglia are more than an “organ of habit” in the brain, as it plays a role in perception, cognition, and emotional behaviors (Jahanshahi et al., 2015).

### tPA-Cer puncta are localized to large MFBs in the stratum lucidum CA3 subregion of the hippocampus

**Figure 6.**
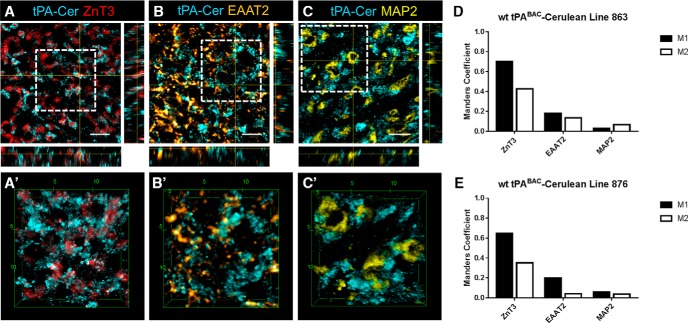
tPA-Cer is localized to large MFBs and astrocytes in the CA3 stratum lucidum lamina of the hippocampus. Subcellular colocalization of tPA in stratum lucidum of the hippocampus was investigated using high-resolution confocal microscopy. Images (63×) are representative regions of interest from the stratum lucidum lamina of tPA^BAC^-Cer transgenic mice and are presented as 5-μm z-stacks (Δz = 0.5 μm) visualized in orthogonal YZ and XZ slices (***A****–****C***) and magnified 3D maximum intensity projections (***A’****–****C’***), for each of the respective colocalization markers. For quantification of colocalization using the Manders coefficient (***D***, ***E***) entire images were concatenated from 5-μm z-stacks (512 × 512 pixels; Δz = 0.5 μm) that were independently acquired two to four times (ZnT3: mice *n* = 10, lines 863 and 876; EAAT2: mice *n* = 6–8, lines 863 and 876, respectively; MAP2: mice *n* = 4, lines 863 and 876). tPA-Cer puncta was found to colocalize with the zinc transporter-3 (ZnT3; red), which has previously been observed exclusively in MFBs, as indicated visually by the white overlay in the orthogonal sections and 3D max projection (Manders M1: 0.704 and 0.649 for lines 863 and 876, respectively; Costes probability *p* value ≥ 95%; ***D***, ***E***). Partial colocalization was weakly observed in astrocytes visualized with the astrocytic glutamate transporter EAAT2 (orange; ***B***, ***B’***), which is in agreement with the lower quantified colocalization coefficient (Manders M1: 0.183 and 0.198 for lines 863 and 876, respectively; Costes probability *p* value ≥ 95%; ***D***, ***E***). To confirm the presynaptic localization of tPA to MFBs, sections were also stained for the dendritic marker MAP2, which detects the dendritic thorny excrescences of CA3 pyramidal neurons that MFBs encase. Orthogonal YZ and XZ slices and 3D max projections of tPA-Cer and MAP2 (yellow; ***C***, ***C’***) showed no colocalization (Manders M1: 0.032 and 0.060 for lines 863 and 876, respectively; Costes probability *p* value = 0.0%; ***D***, ***E***). ZnT3, zinc-transporter 3; EAAT2, excitatory amino acid transporter 2; MAP2, microtubule associated protein 2. Scale bars: 5 μm (***A–C***) and the units on the scale boxes are given in μm (***A’–C’***).

Our previous results established that tPA-Cer fluorescence is highly enriched in the mossy fiber pathway (Figs. 4*D*, 5*G* ). These findings are consistent with immunohistochemical analysis and *in situ* zymography which also report high levels of tPA localization and proteolytic activity in the mossy fiber pathway (Sappino et al., 1993; Salles and Strickland, 2002; Louessard et al., 2016). As mossy fiber axons are known to have morphologically and functionally distinct presynaptic terminals we leveraged the use of the tPA^BAC^-Cer mice, combined with high-resolution confocal imaging, to establish tPA’s subcellular distribution in the hippocampus. Confocal z-stacks were captured of both tPA-Cer and putative colocalization markers. Dorsal hippocampal sections from tPA^BAC^-Cer mice were first probed for zinc transporter-3 (ZnT3), which is the protein correlate underlying the Timm’s histochemical stain to visualize the mossy fiber pathway (Frotscher et al., 1994). ZnT3 was used based on previous electron microscopy immunocytochemistry of ZnT3 in the murine brain which revealed the exclusive localization of ZnT3 to large MFBs (Wenzel et al., 1997). An analysis of orthogonal YZ and XZ sections from a 5-μm z-stack (Δz = 0.5 μm) suggests that tPA-Cer puncta colocalize with ZnT3 (Fig. 6*A*). Further, 3D projection confirms that tPA-Cer puncta reside in ZnT3-positive MFBs (Fig. 6*A’* ). As shown in Figure 6*D*,*E*, quantification of colocalization between tPA and ZnT3 from 5-μm concatenated image stacks (mice, *n* = 10), shows that tPA-Cer has a high degree of overlap with ZnT3 (M1 coefficient: 0.704 and 0.649 for lines 863 and 876, respectively; Costes probability *p* value ≥ 95%). There was no observable detection of cerulean fluorescence in transgene negative littermate controls (Fig. 5*H*). Conversely, ZnT3 shows only partial overlap with tPA-Cer (M2 coefficient: 0.426 and 0.352 for lines 863 and 876, respectively). The lack of a one-to-one relationship between the M1 and M2 coefficients is possibly due to the zinc transporter being a synaptic, not dense core, vesicle marker. Although dense core vesicles are sporadically found in MFBs (Wenzel et al., 1997; Rollenhagen et al., 2007), synaptic vesicles are much more abundant. ZnT3 staining, therefore, likely illuminates a larger area of the MFB, while the tPA-Cer signal, presumably in dense core vesicles, appears more punctate (Silverman et al., 2005; Lochner et al., 2008; Scalettar, et al., 2012). Lastly, these data indicate that while the vast majority of tPA is localized to giant MFBs there is also a population that occupies another locale.

As astrocytes are known to wrap fine processes around MFBs (Rollenhagen and Lubke, 2010) and as tPA has been shown to be taken-up by astrocytes (Cassé et al., 2012), tPA^BAC^-Cer hippocampal sections were stained for the astrocytic glutamate transporter EAAT2 (excitatory amino acid transporter 2). While a strong visual colocalization was difficult to ascertain from orthogonal YZ and XZ slices (Fig. 6*B*) and 3D projections (Fig. 6*B’*), image quantification (Fig. 6*D*,*E*) did demonstrate lower levels of colocalization (M1 coefficient: 0.183 and 0.198 for lines 863 and 876, respectively; Costes probability *p* value ≥ 95%). To further validate our results, tPA^BAC^-Cer sections were stained for the dendritic marker MAP2 (microtubule associated protein 2) as a negative control. In stratum lucidum, MAP2 detects the dendritic thorny excrescences of CA3 pyramidal neurons which are the postsynaptic partner to the MFBs. As predicated, orthogonal YZ and XZ slices of tPA-Cer and MAP2 showed no visual overlap (Fig. 6*C*) and a 3D projection (Fig. 6*C’*) showed that tPA-Cer puncta encapsulate the MAP2-positive dendritic thorny excrescences. Moreover, when quantified (Fig. 6*D*,*E*), no colocalization was found (M1 coefficient: 0.032 and 0.060 for lines 863 and 876, respectively; Costes probability *p* value = 0%).

### tPA is expressed in a subset of SST-positive inhibitory interneurons in SO/alveus of CA1 and CA3 hippocampal subfields

**Figure 7.**
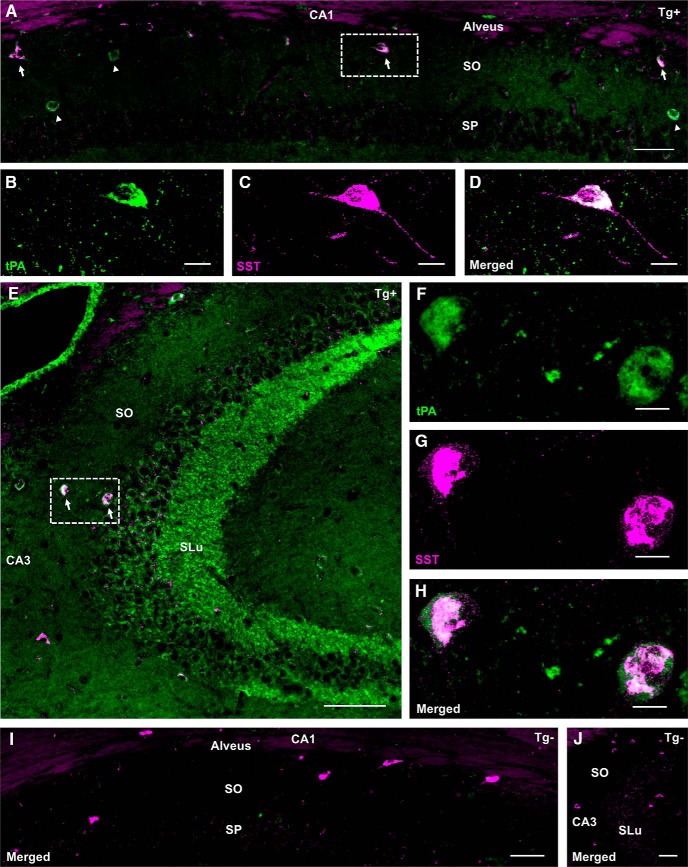
Amplification of tPA-Cer signal reveals a population of cells in SO/alveus of the hippocampal CA1 and CA3 regions that coexpress SST. Representative images of hippocampal CA1 (***A***) and CA3 (***E***) subfields from tPA^BAC^-Cer transgenic mice (mice, *n* = 8) exemplifying the localization and distribution of tPA-Cer cell bodies (tPA; green) that coexpress the inhibitory interneuronal marker SST (magenta). For localization and distribution statistics, see Table 1. With GFP tyramide signal amplification, somatic tPA is also detectable in the pyramidal cell layer of the CA3 subfield (***E***), although it is still largely absent in the CA1 pyramidal cell layer (***A***). Confocal (63×) maximum intensity projections focused on a region of interest (dashed box) highlighting tPA-Cer-positive cells (tPA; green) that coexpress SST (magenta) from the CA1 (***B****–****D***) and CA3 (***F****–****H***) SO/alveus lamina. Unlike the punctate nature of tPA in axonal projections, tPA’s somatic expression appears more diffuse. ***I***, ***J***, Immunostaining for SST and GFP with tyramide signal amplification in transgene negative controls only revealed SST-positive interneurons in the SO/alveus lamina of hippocampal CA1 (***I***) and CA3 (***J***). SLu, stratum lucidum. Scale bars: 50 μm (***A***), 10 μm (***C***, ***D***), 100 μm (***E***), 10 μm (***F–H***), 50 μm (***I***), and 100 μm (***J***).

The presence of sporadic β-Gal puncta in SO, SR, and the hilus (Fig. 2*A*) of the hippocampus in PlatβGAL reporter mice suggested that other cell types, in addition to the granule and pyramidal cells, express tPA. In an effort to visualize and identify these cells in the tPA^BAC^-Cer transgenic mice, the cerulean signal was magnified using a GFP antibody in conjunction with tyramide signal amplification. With amplification, tPA-Cer-positive cell bodies were revealed in the CA1 and CA3 hippocampal subfields of transgene positive mice (Fig. 7*A*,*E*). CA3 pyramidal cells became visible, but more strikingly were the sparsely-populated tPA-Cer-expressing cell somas in SO, SP, and SR, although most were strongly localized to the SO/alveus lamina of the CA1 and CA3 subfields (Table 1). To quantify strata localization of tPA-Cer cell bodies, two to four hippocampal sections per mouse (mice, *n* = 8) for each of the transgenic lines were analyzed. Our results show that for a hippocampal section there were, on average, 11.68 ± 1.53 (*n* = 301 cells) and 10.90 ± 2.06 (*n* = 238 cells) tPA-Cer cells bodies in the CA1 SO/alveus region and 5.58 ± 1.14 (*n* = 141 cells) and 5.34 ± 1.98 (*n* = 115 cells) tPA-Cer cell bodies in the CA3 SO/alveus region for lines 863 and 876, respectively. Less frequently, on average, 3.32 ± 1.15 (*n* = 78 cells) and 2.28 ± 1.06 (*n* = 52 cells) tPA-Cer cells bodies were found in SR and SP for lines 863 and 876, respectively. No statistical difference was noted between lines 863 and 876.

**Table 1.** Localization and coexpression of tPA-Cer-positive soma in hippocampus of tPA^BAC^-Cer mice

	Localization of tPA-Cer-positive soma			Coexpression of tPA-Cer-positive soma	
	CA1 SO alveus	CA3 SO alveus	CA1 and CA3 SP/SR	tPA-Cer-positive neurons expressing SST (%)	SST-positive interneurons expressing tPA (%)
Line 863	11.68 ± 1.53	5.58 ± 1.14	3.32 ± 1.15	54.35 ± 6.32	53.89 ± 8.82
	(*n* = 301)	(*n* = 141)	(*n* = 78)	(*n* = 520)	(*n* = 520)
Line 876	10.90 ± 2.06	5.34 ± 1.98	2.28 ± 1.06	58.90 ± 6.74	44.66 ± 9.45
	(*n* = 238)	(*n* = 115)	(*n* = 52)	(*n* = 405)	(*n* = 540)

Results are presented as the mean ± CI_(0.95)_ for a given hippocampal section. Cell count data were gathered from SO/alveus, SP, and SR; *n* refers to the total number of cells counted from two to four hippocampal sections per tPA^BAC^-Cer transgenic mouse (lines 863 and 876; mice = eight per line).

The sporadic nature of tPA-Cer-positive cell bodies is consistent with that of GABAergic interneurons (Oliva et al., 2000). To test if tPA-Cer-positive cells are indeed GABAergic interneurons, various immuno-markers for SO/alveus-interneurons were used to neurochemically identify the subpopulation of tPA-Cer-positive cells in the hippocampus (Somogyi and Klausberger, 2005). tPA-Cer-positive cells were found to strongly coexpress the interneuronal marker SST in the SO/alveus lamina of hippocampal regions CA1 and CA3 (Table 1; Fig. 7). Widefield images of the hippocampus show prominent tPA expression in stratum luciudum (Fig. 7*E*), but also in SST-positive interneurons scattered throughout SO/alveus (Fig. 7*A*). Immunostaining for SST and GFP with tyramide signal amplification in transgene negative controls only revealed SST-positive interneurons (Fig. 7*I*,*J*). Confocal images from CA1 (Fig. 7*B–D*) and CA3 (Fig. 7*F–H*) show tPA-Cer cells (green) that clearly overlap with SST-positive (magenta) interneurons. When quantified, for a given hippocampal section, on average ∼54.35 ± 6.32% (*n* = 520 cells) and 58.90 ± 6.74% (*n* = 405 cells) of tPA-Cer cells were found to coexpress SST, while 53.89 ± 8.82% (*n* = 520 cells) and 44.66 ± 9.45% (*n* = 540 cells) of SST-positive cells were found to coexpress tPA-Cer, for lines 863 and 876, respectively. No statistical difference was noted between lines 863 and 876.

**Figure 8.**
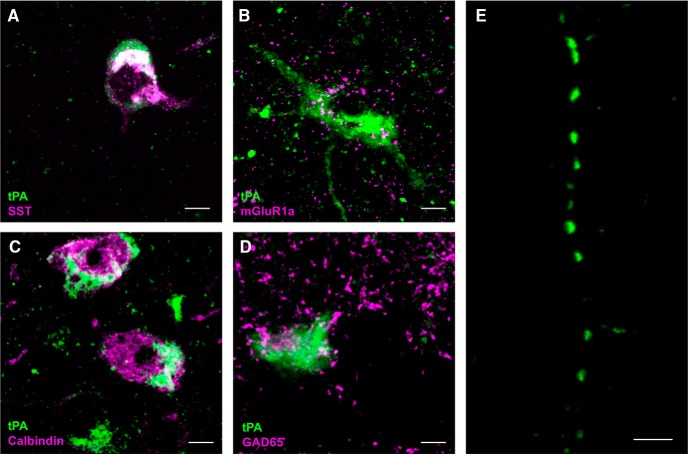
tPA-Cer cells are positive for immunocytochemical markers of O-LM inhibitory interneurons. Dorsal hippocampal sections from tPA^BAC^-Cer transgenic mice were probed for previously confirmed immunocytochemical markers of O-LM inhibitory interneurons. While O-LM interneurons are known for expressing SST (***A***), other morphologically distinct interneuron subgroups have been shown to stain positive for the neuropeptide. O-LM interneurons, however, have been shown to be strongly decorated with the metabotropic glutamate receptor 1a (mGluR1a), and they have been shown to express the calcium-binding protein calbindin. High-resolution, confocal images (63×) of 10-μm-thick z-stack maximum intensity projections demonstrate that tPA-Cer-positive cells bodies in SO/alveus coexpress both mGluR1a (***B***) and calbindin (***C***). Sections were also stained for GAD65, confirming the GABAergic nature of the tPA-Cer-expressing cells (***D***). In agreement with this, O-LM immunocytochemical profile was the observation of axonal-like projections in SR, as axons of O-LM interneurons extend to stratum lacunosum moleculare (***E***). Pictures are representative images from SO/alveus of stainings from at least four transgenic mice (*n* = 4–8) per interneuronal marker. mGluR1a, metabotropic glutamate receptor 1a; GAD65, glutamic acid decarboxylase 65. Scale bars: 5 μm (***A–E***).

Since O-LM interneurons, whose cell bodies reside in SO and send axonal projections to stratum lacunosum-moleculare, are known to express SST (Fig. 8*A*), other neurochemical markers of O-LM interneurons were probed for to see whether tPA-Cer cells can be immunocytochemically classified as O-LM interneurons. tPA-Cer cells were found to coexpress the calcium-binding protein calbindin (Fig. 8*C*), which has previously been reported to comprise roughly 32% of SST/O-LM interneurons (Oliva et al., 2000); they were also found to coexpress the metabotropic glutamate receptor 1 (mGlur1a; Fig. 8*B*), which is highly expressed in SST/O-LM interneurons (Klausberger, et al., 2003; Somogyi and Klausberger, 2005; Sylwestrak and Ghosh, 2012). Interestingly, tPA mRNA polyadenylation and translation has previously been shown to be dependent on mGluR1 activation (Shin et al., 2004). To confirm the inhibitory nature of these cells, immunohistochemistry against GAD65 (glutamic acid decarboxylase 65) in tPA^BAC^-Cer mice was performed (Fig. 8*D*). In agreement with the localization and cytochemical profile of SST/O-LM interneurons, tPA-Cer puncta were observed in structures reminiscent of axonal processes in SR (Fig. 8*E*). These data strongly suggest that at least a portion of tPA-Cer cells can be categorized as O-LM interneurons.

## Discussion

In the present study, we have confirmed and extended our understanding of the expression of tPA in the adult murine brain. Using both a PlatβGAL reporter mouse and a BAC transgenic mouse expressing a tPA-Cer fusion protein, we have provided a detailed characterization of the regional, cellular, and sub-cellular localization of tPA. While largely complimenting the expression pattern observed in transgenic mice that harbored a 9.5-kb segment of the human tPA promoter to drive expression of LacZ (Yu et al., 2001), PlatβGAL reporter mice displayed differences that provide insight into the transcriptional regulation of tPA. In contrast to the PlatβGal reporter mice, in the human 9.5-kb tPA^LacZ^ reporter mouse, β-Gal staining was only weakly observed in the CA3 subfield, and there was no detection of β-Gal in blood vessels, a well-established site of tPA expression (Fredriksson et al., 2015; Louessard et al., 2016). In addition, Yu et al. (2001) observed high tPA/LacZ expression in the medial habenula, which was not the case for the PlatβGAL reporter mice. These discrepancies suggest that regulatory elements important for the regional and cellular expression patterning of tPA are not encompassed in the 9.5-kb human promoter segment or that there are differences between the human and murine promoter sequences that do not completely recapitulate species specific expression of tPA.

When comparing the PlatβGAL reporter mice and the tPA^BAC^-Cer transgenic mice, there is a clear uncoupling between where tPA is synthesized and where it is trafficked, which is in agreement with previous *in situ* expression studies examining tPA mRNA and tPA-catalyzed proteolysis (Sappino et al., 1993). And, while the laminar, tri-synaptic circuitry of the hippocampus illustrates this uncoupling most distinctly, tPA’s differential expression pattern is also apparent in the amygdala and the basal ganglia. In addition, there is a stark dichotomy between β-Gal expression and tPA-Cer fluorescence in the cortex and cerebellum. Although β-Gal is abundant throughout the cortex and cerebellum in PlatβGAL reporter mice, other than faintly positive cell bodies in the piriform and entorhinal cortex, there is no detectable tPA-Cer fluorescence in the cortex and cerebellum of tPA^BAC^-Cer transgenic mice. Tracing experiments of cortical and cerebellar projections, which are beyond the scope of this article, would help address this discrepancy. For, if tPA is primarily trafficked as our data suggests, then it is possible that tPA-Cer is localized along cortical descending pathways or in efferent targets in the cerebellum, the basal ganglia, the brainstem, and spinal cord. Similarly, more detailed tracing studies would be required to assess if tPA-Cer is localized to cerebellar efferents, like the vestibulocerebellum, spinocerebellum, and cerebrocerebellum pathways, and their target nuclei. Given that tPA’s site of action is removed from its site of synthesis, the tPA^BAC^-Cer transgenic mice, when analyzed in conjunction with the PlatβGAL reporter mice, provide a more informative expression profile of tPA in the adult murine brain.

Taking advantage of the tPA-Cer fusion construct, therefore, we report for the first time tPA’s subcellular localization to giant MFBs in stratum lucidum of CA3. Previous studies have only generally described tPA expression in the mossy fiber pathway, without examining its specific compartmentalization. The specific structural localization can potentially provide meaningful insight into tPA’s function. This is especially true since mossy fiber axons of DGCs display two other morphologically distinct presynaptic terminals, small *en passant* boutons and filipodial extensions that emanate from the MFBs (Acsády et al., 1998; Rollenhagen and Lübke, 2010). Moreover, these structurally distinct terminals have divergent postsynaptic targets; MFBs synapse with hilar mossy cells and the apical dendritic spines or “thorny excrescences” of CA3 pyramidal cells, while *en passant* boutons and filipodial extensions preferentially target GABAergic interneurons in the hilus and stratum lucidum (Frotscher et al., 1994; Acsády et al., 1998). The mossy fiber-to-CA3 pyramidal cell synapse also has a different synaptic physiology; compared to the mossy fiber-to-interneuron synapse, the mossy fiber-to-CA3 pyramidal cell synapses shows marked paired-pulse facilitation and LTP (Salin et al., 1996; Henze et al., 2000; Toth et al., 2000; Nicoll and Schmitz, 2005).

Thus, the specific localization of tPA-Cer puncta to giant MFBs suggests that it may have a role in regulating synaptic efficacy at the mossy fiber-to-CA3 pyramidal cell synapse. Consistent with this model, functional studies demonstrate tPA^-/-^ mice to have deficits in LTP in the mossy fiber pathway (Huang et al., 1996). Further interrogations into the mechanism underlying deficits in LTP have focused on a postsynaptic locus of expression. Mice deficient in tPA, which carries a cAMP response element in its promoter, exhibit reduced potentiation by cAMP analogs (Huang et al., 1996); blocking tPA’s non-proteolytic interaction with the postsynaptically expressed low-density lipoprotein receptor-related protein (LRP) causes deficits in synaptic potentiation (Bu et al., 1992; Zhuo et al., 2000); and tPA-mediated cleavage of proBDNF was found to be essential for the full expression of LTP (Korte et al., 1995, 1998; Pang et al., 2004). These mechanistic studies, however, presumed postsynaptic tPA expression and were performed in the hippocampal CA1 region, not at the mossy fiber-to-CA3 pyramidal cell synapse, where we have shown tPA to be most highly expressed in the giant MFBs. Additionally, while amplification of the tPA-Cer signal was able to reveal tPA expression in the soma of CA3 pyramidal cells, high-resolution colocalization analysis did not uncover tPA in the postsynaptic thorny excrescences of CA3 pyramidal cells. In fact, no colocalization was observed between tPA-Cer puncta and the dendritic marker MAP2. Although *in vitro* studies have shown activity-dependent release of tPA from both pre- and postsynaptic compartments (Gualandris et al., 1996; Lochner et al., 1998, 2006; Scalettar et al., 2012), our *in situ* expression data demonstrates that tPA’s localization is largely presynaptic and suggests that potential presynaptic neuromodulatory effects at the mossy fiber-to-CA3 synapse may have been overlooked.

Interestingly, tPA-Cer puncta were also observed to partially colocalize with astrocytes. It is unclear, however, if the tPA present is endogenous to astrocytes, as transcriptome analysis has shown astrocytes to express tPA mRNA *in vitro* (Zhang et al., 2014), or if tPA is endocytosed by astrocytes in an LRP-dependent fashion (Cassé et al., 2012). The mossy fiber-to-CA3 pyramidal cell synapse appears unique with respect to its trisynaptic cytoarchitecture. Previous studies indicate that astrocytes completely insulate the synapse, cordoning off the active zone and synaptic cleft from the surrounding parenchyma, but not physically encroaching into the cleft (Rollenhagen et al., 2007). And, while there is no *in vivo* functional evidence demonstrating the effects of tPA release from astrocytes on synaptic function, *in vitro* evidence has pointed to tPA acting as a gliotransmitter that is released and recycled by astrocytes (Cassé et al., 2012). The lack of perisynaptic astrocytic processes making contact with axon-spine interfaces (Rollenhagen et al., 2007), however, possibly indicates that, at least at the mossy fiber-to-CA3 pyramidal cell synapse, diffusion-limited gliotransmitter tPA may have reduced effects on synaptic function. Moreover, if tPA is released in an activity-dependent manner (Gualandris et al., 1996; Parmer et al., 1997) from giant MFBs, the configuration of the mossy fiber-to-CA3 pyramidal cell synapse suggests that it is not geared toward immediate clearance and uptake, but rather, toward potentiating the effects of tPA and presumably enhancing synaptic efficacy.

In this study, we also identify tPA expression in a subset of SST-positive interneurons in the hippocampal SO/alveus lamina. We provide a detailed distribution of tPA-Cer-positive cell bodies in both the CA1 and CA3 subfields and their quantified coexpression with SST-positive interneurons. While enhancement of the tPA-Cer protein, with anti-GFP tyramide amplification, was necessary to distinctly visualize these cell bodies, we believe this somatic expression of tPA is physiologic as (1) no signal was observed in littermate transgene negative controls, (2) the recapitulation of tPA expression in the mossy fiber pathway indicates that tPA-Cer is appropriately targeted to its cellular and subcellular locale, and (3) β-GAL puncta in the PlatβGAL reporter mice are noticeable in SO/alveus and SR (Fig. 2). Presumably, the increase in tPA expression, due to extra copy number from the BAC transgene, allowed for the detection of a previously unrecognized and specific population of tPA-expressing cells. It is unclear, however, why tPA is differentially localized to the soma or axonal projection and if such differential trafficking is functionally significant. For, tPA appears to be largely localized to the soma of inhibitory interneurons (Fredriksson et al., 2015) and to the axons of excitatory neurons (Louessard et al., 2015). While knowledge about dense core vesicle trafficking is still nascent compared to synaptic vesicle trafficking, there is evidence which demonstrates regionally-specific, differential trafficking of the neuropeptide NPY to dendrites and axons (Ramamoorthy et al., 2011) and that excitatory and inhibitory neurons in the hippocampus exhibit different dense core vesicle molecular machinery (Ramírez-Franco et al., 2016).

In the CA1 hippocampal region alone, more than a dozen different types of interneurons have been classified based on their morphologic, neurochemical and physiologic properties (Freund and Buzsáki, 1996; Somogyi and Klausberger, 2005). And, while no one single marker is indicative of a specific type of interneuron, tPA-Cer-positive cells appear to share a very similar somatic distribution (Oliva et al., 2000) and immunocytochemical profile with O-LM interneurons (Somogyi and Klausberger, 2005; Minneci et al., 2007; Sylwestrak and Ghosh, 2012). As tPA-expressing neurons have never been described in SST/O-LM interneurons before, it is unclear how tPA may be exerting its effects. Morphologically, the axonal projections of O-LM interneurons ramify at the distal apical dendrites of CA1 pyramidal cells, where perforant path fibers from the entorhinal cortex terminate. Functionally, O-LM interneurons are known to fire rhythmically at the trough of theta (4-8 Hz) oscillations in the hippocampus (Klausberger et al., 2003), and they have been shown to facilitate LTP in the Schaffer collateral-to-CA1 pathway. Although deficiency in tPA has been previously implicated in defects in the late-phase of LTP in the hippocampal CA1 region (Huang et al., 1996; Calabresi et al., 2000), the contribution of tPA from O-LM interneurons has not been specifically tested in this paradigm. Plasticity of glutamatergic CA1 synapses onto O-LM interneurons has also been investigated, as changes in synaptic efficacy may have an important role in modulating network excitability (Nicholson and Kullmann, 2014). To date, however, it is unknown if tPA is involved in these events.

Taken together, the regional, cellular, and subcellular characterization of tPA expression presented here provides a primer on tPA’s role in the CNS. Many of the foundational experiments on tPA’s function in the brain were performed before a detailed description of its protein localization, this is especially confounding in the case of tPA as its site of synthesis is uncoupled from it targeted site of action. With the generation of the tPA^BAC^-Cer transgenic mice and its appropriately targeted tPA-Cer fusion protein, however, future mechanistic studies to elucidate tPA’s function are now possible.

## Synthesis

Reviewing Editor: Jun Chen, University of Pittsburgh Decisions are customarily a result of the Reviewing Editor and the peer reviewers coming together and discussing their recommendations until a consensus is reached. When revisions are invited, a fact-based synthesis statement explaining their decision and outlining what is needed to prepare a revision will be listed below. The following reviewer(s) agreed to reveal their identity: Manuel Yepes, Tanuj Gulati. Note: If this manuscript was transferred from JNeurosci and a decision was made to accept the manuscript without peer review, a brief statement to this effect will instead be what is listed below.

Both reviewers acknowledged the potential importance of your findings. However, they raised a number of concerns that need to be clarified.

Reviewer #1:

This is a review on a paper that seeks to characterize the expression and trafficking of tissue type plasminogen activator in the murine brain. This is an interesting study that uses a very elegant approach to confirm many aspects of what is already known about the expression and trafficking of tPA in the brain. However, the authors also describe some few provocative and very interesting differences with previous reports by other groups that may lead to a better understanding of tPA's function in the brain.

Abstract:

1. Blood brain barrier leakage should be replaced for BBB permeability

2. Seizure should be changed for seizure spreading, or seizures.

3. The authors cannot state that the :“molecular mechanisms of tPA's action remains unclear”, as over the last 20 years a large number of publications from many groups have addressed the molecular mechanism of tPA's action.

Significance statement

1. The sentence “little is known about the in situ expression and localization of tPA...” is not accurate, as several groups have published reports on that topic.

Introduction

1. First sentence: it has long been known that tPA is also expressed in neurons, and perhaps some sub-population of glia.

2. Line 5: it is now clear that the reason for rtPA not to be given beyond 4.5 hours of onset of symptoms is not the increased risk of hemorrhagic conversion as much as its lack of thrombolytic efficacy beyond that time point.

3. The introduction should also state that several studies have found that tPA also has a direct effect on synaptic function.

Results

1. The authors state that males and females were used for their studies. It will be interesting and very novel to know whether there are sex-dependent differences in the expression of tPA in the brain.

2. At the magnification shown in figure 1 is impossible to visualize B-gal expression in the blood vessels and in cortical layers II-VI as the authors describe it in the manuscript.

3. Is there a mismatch between B-gal and tPA immunoreactivity in the cerebral cortex, or only in the hippocampus?

4. What is the pattern of tPA immunoreactivity in the cerebral cortex?

5. How is the tPA-Cer expression in the cerebral cortex?

Discussion

1. In page 27 the authors state that astrocytes completely insulate the synapse thus that in the mossy fiber-to-CA3 pyramidal cell synapse the anatomical configuration of the astrocyte-neuron interaction does not support a model in which astrocytes re-uptake tPA from the synapse. This is not accurate. The authors should consider and discuss the fact that the anatomy of the interaction between the synapse and perivascular astrocytes is highly dynamic and continuously changes in response to synaptic activity, and that in active synapses that coverage is incomplete or even absent.

2. At some points the discussion is too speculative.

Figures:

1. Figure 6 lacks statistical analysis. How the ROI were chosen? Some of the micrographs show large un-stained spaces which may affect the final quantification. Furthermore, statistical analysis should be performed with one-way ANOVA with a post-hoc analysis to identify the highest coefficients. Also, a number should be provided in the figure for the scale bars.

Reviewer #2:

This is a nice study that profiles tPA in the murine brain, and showcases its differential expression in hippocampal-limbic areas. They show how their study points that tPA localization is differentially regulated, and might have a bearing on its neuromodulatory effects.

I would want the authors to expand the discussion on the physiologic effects of their findings, especially with their finding of tPA expression in SST interneurons in hipppocampal stratum oriens region. Are there any specific LTP/ paired pulse facilitation studies that authors can cite in this region, perhaps also with tPA knock out mice, so that readers can have some insight into functional implications of this important finding.

## Author Response

We were grateful to the reviewers’ for their insightful comments and have revised the manuscript to address all of the points raised. Please find below specific responses to each comment and in the manuscript sections with significant revisions are now highlighted in yellow.

Specific responses to the reviewers’ points

Reviewer #1:

This is a review on a paper that seeks to characterize the expression and trafficking of tissue type plasminogen activator in the murine brain. This is an interesting study that uses a very elegant approach to confirm many aspects of what is already known about the expression and trafficking of tPA in the brain. However, the authors also describe some few provocative and very interesting differences with previous reports by other groups that may lead to a better understanding of tPA's function in the brain.

Abstract:

1. Blood brain barrier leakage should be replaced for BBB permeability

We have changed BBB leakage to “BBB permeability.”

2. Seizure should be changed for seizure spreading, or seizures.

We have changed seizure to “seizure progression.”

3. The authors cannot state that the :“molecular mechanisms of tPA's action remains unclear”, as over the last 20 years a large number of publications from many groups have addressed the molecular mechanism of tPA's action.

We have changed the wording to address this concern.

Significance statement:

1. The sentence “little is known about the in situ expression and localization of tPA...” is not accurate, as several groups have published reports on that topic.

We have changed the wording to address this concern.

Introduction:

1. First sentence: it has long been known that tPA is also expressed in neurons, and perhaps some sub-population of glia.

While we agree that tPA is also known to be expressed by neurons, tPA is still more well-known for its role in fibrinolysis. As such, we decided to first introduce tPA for its established proteolytic function in the vasculature and then transition to its role in the central nervous system, which is more complex.

2. Line 5: it is now clear that the reason for rtPA not to be given beyond 4.5 hours of onset of symptoms is not the increased risk of hemorrhagic conversion as much as its lack of thrombolytic efficacy beyond that time point.

We thank Reviewer 1 for stressing this point and have made the appropriate changes to address the reviewer's comment.

3. The introduction should also state that several studies have found that tPA also has a direct effect on synaptic function.

We specifically cite the earliest papers demonstrating a role for tPA in modulating synaptic efficacy (Frey et al., 1996) and the late-phase of LTP (Huang et al., 1996) in paragraph 2 of the Introduction. We have also added a more recent study demonstrating changes in miniature excitatory post-synaptic currents (mEPSCs) in CA1 pyramidal neurons upon application of tPA (Wu et al., 2015).

Results:

1. The authors state that males and females were used for their studies. It will be interesting and very novel to know whether there are sex-dependent differences in the expression of tPA in the brain.

While we cannot exclude the possibility that there may exist some sex-dependent differences of tPA expression in the adult murine brain, we did not detect any gross disparities. The bed nucleus stria terminalis (BNST) is reported to be sexually dimorphic in humans (Allen and Gorski, 1990; Lebow and Chen, 2016), but to our knowledge, such sex-dependent differences in size of the BNST have not been studied in mice. In our limited survey through the adult murine brain we did not detect any differences in tPA-Cer expression in the BNST between male and female mice. We also did not measure any differences between male and female mice in the Mander's coefficient for co-localization of tPA-Cer and ZnT3, tPA-Cer and EAAT2, or tPA-Cer and MAP2.

Huang et al. (1996) did report sex-dependent differences between male and female tPA-/- mice in fear conditioning. Female tPA-/- mice froze significantly longer than their female wild-type controls in both context and cued conditioning, whereas male tPA-/- mice were not significantly different than their male wild-type controls. These studies have not been repeated, however. Moreover, the genetic background of the mice was not reported, nor was it reported whether or not littermates were used as controls. To our knowledge there have been no other reported sex-dependent differences observed regarding tPA function and/or localization.

2. At the magnification shown in figure 1 is impossible to visualize B-gal expression in the blood vessels and in cortical layers II-VI as the authors describe it in the manuscript.

We thank the Reviewer for this observation and agree that it may be difficult for the readers to appreciate β-Gal expression in the blood vessels. We no longer make reference to blood vessels in Figure 1 in the main text or figure text and we have removed the arrowheads in Figure 1. We now instead only refer to β -Gal expression in blood vessels in Figure 2, which is shown at a higher magnification, making the vessel localization more apparent.

3. Is there a mismatch between B-gal and tPA immunoreactivity in the cerebral cortex, or only in the hippocampus?

As the hippocampus has served as the model system for studying tPA's role in synaptic plasticity, we focused our high-resolution imaging and colocalization and co-expression studies on the hippocampus. However, when probing for tPA in the hippocampus of heterozygous PlatβGal reporter mice (Bregma -2.0mm), we were able to assess certain cortical regions for expression of tPA. In these cases we did not observe any tPA immunoreactivity in cells bodies or nerve fibers in the retrosplenial, motor, somatosensory, auditory, entorhinal, or piriform cortex in heterozygous PlatβGal reporter mice.

Though we did not specifically examine an uncoupling between tPA's site of synthesis and its site of trafficking outside the hippocampus in PlatβGal mice, the differential expression of β-Gal and tPA-Cer is apparent when comparing the serial images from the PlatβGal reporter mice and the tPABAC-Cer transgenic mice. For example, while β-Gal expression is present in both the basolateral (BLA) and centromedial amygdala (CeA and MeA), tPA-Cer is only expressed in the centromedial amygdala. A similar delineation is also apparent in the basal ganglia. β-Gal is present in the caudate/putamen, but absent in the external and internal segments of the globus pallidus. In contrast, tPA-Cer is absent in the caudate/putamen, but present in the globus palldius. Both of these expression patterns in the amygdala and basal ganglia are consistent with tPA being trafficked to axon fibers.

Therefore, we have elaborated on this differential expression pattern in the main text and we have included the NeuN stain of the higher-magnification tPA-Cer positive regions in Figure 4 to demonstrate that tPA is predominantly localized to the nerve fibers in these regions, not the cell bodies. Though that is not to say there are no tPA-Cer cell bodies in the cortex, as we did observe tPA-Cer expression in the piriform and entorhinal cortex of tPABAC-Cer transgenic mice (Figure 4).

4. What is the pattern of tPA immunoreactivity in the cerebral cortex?

As discussed above, the focus of our study was the hippocampus; however, as also indicated above, we do see a similar disconnect between sites of expression and the trafficked tPA. Since it is much harder to identify specific sources of axonal tracts in the cortex we believe that it is appropriate for this study to maintain our focus on the hippocampus for the detailed high resolution study while only presenting a broader survey of the cortex and cerebellum.

5. How is the tPA-Cer expression in the cerebral cortex?

We did not observe tPA-Cer expression in the cerebral cortex in cell bodies, other than faint tPA-Cer positive cell bodies in the piriform and entorhinal cortex (Figure 4).

Discussion

1. In page 27 the authors state that astrocytes completely insulate the synapse thus that in the mossy fiber-to-CA3 pyramidal cell synapse the anatomical configuration of the astrocyte-neuron interaction does not support a model in which astrocytes re-uptake tPA from the synapse. This is not accurate. The authors should consider and discuss the fact that the anatomy of the interaction between the synapse and perivascular astrocytes is highly dynamic and continuously changes in response to synaptic activity, and that in active synapses that coverage is incomplete or even absent.

We appreciate Reviewer 1's concerns with our discussion of the cytoarchitecture of the mossy fiber-to-CA3 synapse and how that architecture might influence tPA's effects on synaptic function. We did not mean to imply that tPA release and clearance by astrocytes does not occur at the mossy fiber-to-CA3 synapse. But given the ultrastructure of the mossy fiber-to-CA3 synapse, where the synaptic cleft is completely isolated from perisynaptic astrocytic processes, we were merely speculating on what this unique architechture could possibly indicate about astrocytic tPA release and clearance and whether these processes can effect synaptic function

We agree with Reviewer 1 that it is important to consider the dynamic nature of astrocytes and how the ultrastructure of the tripartite synapse can change with synaptic activity (Bernardinelli et al., 2014; Genoud et al., 2006; Theodosis et al., 2008; Allen et al., 2017). These structural plasticity studies, however, were focused on synapses where astrocytes are intimately associated with the synaptic cleft. In contrast, astrocytes at the mossy fiber-to-CA3 synapse are hundreds, not tens, of microns away from the synaptic cleft. Therefore, we believe that it is not clear what the biological reasons and/or consequences of this difference might mean? While our paper doesn’t seek to answer this question, we do speculate that 1) the clearance of tPA from the synapse, if diffusion limited, would be temporally extended, potentiating its effects, and 2) gliotransmitter tPA, if diffusion limited, might have reduced effects on synaptic efficacy at the mossy fiber-to-CA3 synapse. Taking Reviewer 1's concerns into consideration, we have modified the discussion to make this point more clear.

2. At some points the discussion is too speculative.

We appreciate the Reviewer's concerns about the Discussion being too speculative. We have qualified our statements to more fully indicate their conjectural nature.

Figures:

1. Figure 6 lacks statistical analysis. How the ROI were chosen? Some of the micrographs show large un-stained spaces which may affect the final quantification. Furthermore, statistical analysis should be performed with one-way ANOVA with a post-hoc analysis to identify the highest coefficients. Also, a number should be provided in the figure for the scale bars.

The ROIs detailed in sub-panels A’, B’ and C’ for figure 6 were not meant to illustrate the regions that were chosen for co-localization analysis. We apologize for the confusion and have clarified in the main text and caption. The zoomed-in ROIs are only meant to help the reader visualize the images.

As described in detail in the Methods section, co-localization analysis was performed from concatenated images of 5uM thick z-stacks (512 x 512 x 0.5um) that were independently acquired 2 - 4 times (ZnT3: mice n = 10, lines 863 and 876; EAAT2: mice n = 6 - 8, lines 863 and 876, respectively; MAP2: mice n = 4, lines 863 and 876). The images in panels A, B, and C are only of representative regions from the entire image for easier viewing for the reader. We have clarified this in the caption and main text. The scale bar in panels A, B, and C is described in the caption as measuring 5um.

While we appreciate the concern Reviewer 1 has with the “large, unstained sections,” these areas do not affect the co-localization analysis. Manders coefficient, which is based on Pearson's correlation coefficient but with average intensity values taken out of the equation, is not affected by areas devoid of signal. Manders coefficients provide an indication of overlap, and can be a more sensitive measure of co-localization when there large difference in pixel intensity between samples, which is a problem we encountered with Cerulean being a weak fluorophore. Two coefficients are given for Manders: M1 and M2. M1 is the ratio of the “summed intensities of pixels from the green images for which the intensity in the red channel is above zero.” M2 is similarly defined but for the red channel. Therefore, only coincident pixel values and the summed pixel values for each respective channel are factored into Manders coefficient. Moreover, analyzing through the z-stack over the entirety of the image allows for a more unbiased quantification of co-localization. Taking a discrete ROI or a line-scan through a particular area can be subjective.

With respect to Reviewer 1's concerns about the statistical analysis of co-localization we refer to tutorial reviews by Bolte and Cordelieres (2006) and Dunn et al., (2011), and to the original paper describing the mathematical principals and algorithms used by the JACoP software (Costes et al., 2004). Briefly, though, we would like to reiterate that all statistical analysis for colocalization was done using JACoP in FIJI. In addition to the JACoP program calculating the Manders coefficients, Costes randomization control was run for a statistical assessment of whether the calculated colocalization events (tPA-Cer vs ZnT3; tPA-Cer vs MAP2; and tPA-Cer vs EAAT2) occurred by chance. Costes is calculated by comparing the coincidence of colocalization in an original image against the coincidence of colocalization in a randomized imaged of shuffled pixels (200 times). Costes approach is expressed as a percentage; a Probability (P)-value of {greater than or equal to} 95% suggests that colocalization is significant and not random. This is a well-respected and accepted method of imaging analysis. Moreover, as the measured variable (tPA-Cer vs ZnT3; tPA-Cer vs MAP2; and tPA-Cer vs EAAT2) is different for each group, a 1-way ANOVA with a post-hoc test is not appropriate.

Reviewer #2:

This is a nice study that profiles tPA in the murine brain, and showcases its differential expression in hippocampal-limbic areas. They show how their study points that tPA localization is differentially regulated, and might have a bearing on its neuromodulatory effects.

I would want the authors to expand the discussion on the physiologic effects of their findings, especially with their finding of tPA expression in SST interneurons in hipppocampal stratum oriens region. Are there any specific LTP/ paired pulse facilitation studies that authors can cite in this region, perhaps also with tPA knock out mice, so that readers can have some insight into functional implications of this important finding.

We thank Reviewer 2 for their interest in this important finding. Given the numerous studies implicating tPA in the late-phase of LTP in the CA1 region of the hippocampus, it is intriguing to speculate on whether or not tPA in O-LM interneurons is modulating that signal. We have provided some more information about what has been studied regarding O-LM interneurons and LTP in the Schaffer collateral-to-CA1 pathway and LTP in the CA1 pyramidal neurons that synapse on O-LM interneurons. However, to our knowledge, there are no studies directly addressing tPA in O-LM synaptic transmission or synaptic plasticity.
